# The Repair Manual of a Fruit Fly Brain

**DOI:** 10.3390/ijms27156795

**Published:** 2026-07-29

**Authors:** Isabella Peetz, Ayelet Blum, Shawn Ahern-Djamali, Grace Boekhoff-Falk

**Affiliations:** Department of Cell and Regenerative Biology, School of Medicine and Public Health, University of Wisconsin-Madison, 1111 Highland Avenue, Madison, WI 53705, USA; ipeetz@wisc.edu (I.P.); blum7@wisc.edu (A.B.); aherndjamali@wisc.edu (S.A.-D.)

**Keywords:** neurogenesis, gliogenesis, neural regeneration, *Drosophila*

## Abstract

The brain is a complex organ; its diverse functions and plasticity correspond with an intricate structure. Integrating sensory inputs with internal physiological states, the brain orchestrates balance, posture, movement, speech, emotions, the creation of memories, and the ability to learn. Whether due to trauma, disease, or stroke, disruptions to brain architecture have long-lasting consequences for a person’s physical, behavioral, emotional, and cognitive health. Comparative studies using model systems to identify meaningful therapies and treatments are critical to elucidate the mechanisms underlying regenerative processes in the brain. This review focuses on the model organism *Drosophila melanogaster*, which has a large repertoire of available molecular and genetic tools for investigation of neural regeneration.

## 1. Introduction

Neurodegenerative conditions affect millions of people worldwide and represent an important and growing cause of functional decline, long-term disability, and mortality [1]. Three leading causes of neurodegeneration are Alzheimer’s disease (AD), Parkinson’s disease (PD), and traumatic brain injury (TBI). In 2022, an estimated 416 million people worldwide were living across the AD continuum, encompassing individuals with preclinical AD, prodromal AD, and AD dementia. This number represents ~20% of the global population aged 50 and older [2]. Estimates from 2019 included more than 8.5 million individuals diagnosed with PD globally [3,4]. Moreover, the global incidence of TBI recently was estimated at ~21 million annually [5]. While these numbers represent people directly afflicted with these conditions, their impacts are felt much more broadly when one considers interpersonal relationships, the stresses on care providers, and the increases in healthcare demand and spending.

To prevent and treat neurodegenerative disorders, there remains a need to better understand the molecular mechanisms underlying the regeneration of both neurons and glia. In this review, we define ‘neurogenesis’ as the generation of new neurons, and use ‘gliogenesis’ to describe the generation of new glial cells. We recognize that some authors refer to the generation of either neurons or glia as ‘neurogenesis’, most likely because some glia share precursors with neurons during development in vertebrates as well as in *Drosophila*. However, we make the distinction because neurogenesis and gliogenesis represent different aspects of regeneration, with glial cells having unique roles in cleaning up cellular debris, providing scaffolding, improving signaling, and participating in immune responses [6,7]. We also note that gliogenesis is common during homeostasis of the vertebrate brain while neurogenesis is not [8,9].

A variety of laboratory models, including non-human primates, mice, zebrafish, *Drosophila melanogaster*, *C. elegans*, and cultured mammalian neural stem cells, continue to provide insights into both neural degeneration and neural regeneration [10,11,12,13,14,15]. As in other regeneration models, injury can be used as a tool in the nervous system to initiate a regenerative response. This provides controlled spatial and temporal systems in which to study how specific tissues respond to damage, including relevant cellular and molecular mechanisms. Injury models allow researchers to investigate the importance of cell activation, cell cycle progression, blastema formation, morphogenesis, and wound healing during regeneration [16]. Additionally, studying the pathways involved in injury responses, including scarring and regeneration, can help us understand the complex processes that ensue upon tissue damage. Ultimately, injury models help bridge the gap between basic science and clinical applications, offering insights into how organisms repair themselves and how these processes can be harnessed for regenerative medicine.

Here we focus on neural repair in the genetic model organism, the fruit fly *Drosophila melanogaster*. Fruit flies have been a widely used as a valuable model organism for more than a century, possessing homologs of at least 75% of genes associated with human disease [17]. *Drosophila* also share canonical neurotransmitters with vertebrates, including dopamine, gamma aminobutyric acid (GABA), glutamate, acetylcholine, and serotonin, along with octopamine, which is functionally equivalent to human norepinephrine [18]. Because of their similar functions in learning and memory, the *Drosophila* mushroom body has been analogized to the mammalian hippocampus [19]. Differences in the glia-to-neuron ratio in *Drosophila* were originally thought to preclude useful comparisons to the human brain; however, recent studies revealed that the glia:neuron ratio of roughly 2:10 in much of the *Drosophila* brain, and as high as 6:10 around the mushroom body [20], is more similar than initially thought to the widely accepted 1:1 glia-to-neuron ratio in humans [21]. Additionally, the *Drosophila* brain has orders of magnitude fewer neurons (~1.4 × 10^5^) [22,23] than the human brain (~8.6 × 10^10^) [24], making certain types of experiments, including those using whole brains, feasible that would be impossible in more complex systems.

Parallels between developmental and homeostatic pathways in the nervous systems of *Drosophila* and mammals lend further support to the idea that neural regeneration research in *Drosophila* is relevant to humans [25]. Conserved signaling pathways first identified in *Drosophila*, such as Hedgehog and Wnt/Wingless, play critical roles in neurogenesis in both vertebrates and invertebrates [26]. These pathways function in neuronal progenitor cells and their derivatives across species [27]. Janus kinase–Signal Transducers and Activators of Transcription (JAK-STAT) signaling, which can activate cell proliferation in response to extracellular triggers, is also conserved [28], as is the Hippo pathway that plays a prominent role in growth control [29]. The similarities in both genome and anatomy between *Drosophila* and humans along with the reduced complexity of *Drosophila*, facilitate a breadth of nervous system research. In scenarios where using a mammalian model is impractical or difficult, *Drosophila melanogaster* is a powerful alternative.

## 2. Homeostasis and the Response to Neural Injury in Adult Mammals

Our understanding of neurogenesis in adult mammalian nervous systems continues to evolve. Although early studies concluded that the mammalian brain is incapable of producing new neurons after birth [30,31], subsequent work demonstrated adult neurogenesis in multiple regions of the rodent brain [32,33,34,35]. It is now widely accepted that homeostatic neurogenesis occurs in at least two regions of the adult mammalian brain: both the subventricular zone (SVZ) of the lateral ventricles and the subgranular zone (SGZ) in the dentate gyrus (DG) of the hippocampus are sites of adult progenitor cell division and postnatal neurogenesis [36,37,38]. Neurogenesis has also been reported in the human adult dentate gyrus [39].

The low levels of cell proliferation that occur in the healthy, mature mammalian central nervous system provide an opportunity to explore mechanisms involved in both the homeostatic generation of new neurons and glia in adults as well as in response to damage. After a neural injury, there are two commonly recognized phases of damage. The initial phase or ‘primary injury’ is followed by the ‘secondary injury’ [40,41]. Primary injury occurs at the moment of injury; for example, a blow to the head could lead to tissue tearing or crushing of neurons and glia and include damage to the blood–brain barrier. Secondary injury includes a chain reaction of cellular and molecular responses that can worsen the damage. For instance, inflammation can prevent brain tissue from receiving needed nutrients, leading to further cell death. Injuries also lead to the production of reactive oxygen species causing oxidative stress and disruption of the mitochondrial electron transport chain which can also lead to cell death or otherwise compromise function [42]. How these distinct phases of injury affect regeneration remains somewhat unclear but appears to be context dependent. For example, a transient inflammatory response can be beneficial, while a prolonged inflammatory response appears to be harmful [43,44,45].

Following severe damage, reactive astrocytes proliferate and form glial scars [46]. Glial scar formation initially was thought to exacerbate neural damage via the release of inflammatory factors and the blocking of synaptogenesis. However, subsequent studies demonstrated that scars protect unmarred neural tissue from secondary damage [47]. Other proposed benefits of glial scars include sealing the injured tissue to prevent the damage from spreading, controlling cerebral blood flow, regulating immunomodulation, and provoking neurogenesis [48].

During development, astrocytic glia not only provide support for neural circuits, but also serve as phagocytes, clearing pruned synapses and neural debris during the widespread cell death occurring in normally developing nervous systems [49]. Following an injury, the post-injury microenvironment plays a pivotal role in the cellular reaction. The persistence of debris after injury requires glia to clean up the affected tissue. Reactive glia also produce a wide variety of proteins, including growth factors and cytokines, that are important for regeneration [50]. Thus, the coordination of neurogenesis and gliogenesis is essential to regeneration following brain or spinal cord injury.

## 3. Neural Development in *Drosophila*

Despite their relatively recent emergence as models for neural regeneration, the use of *Drosophila* central and peripheral neural structures is powerful due to the accessibility to physical manipulation, the abundant genetic tools, and an extensive and detailed understanding of *Drosophila* neural development. During embryogenesis, neural progenitors (neuroblasts, NBs) divide asymmetrically, in most cases both renewing themselves and producing ganglion mother cells (GMCs) before giving rise to neurons and glia [51,52,53]. Under abnormal conditions, including in animals carrying mutations affecting cell fate or polarity, GMCs can deviate from their normal programs with aberrant numbers of divisions and defects in differentiation of their daughter cell lineages.

At the end of embryonic neurogenesis, NBs in the *Drosophila* larvae face either apoptosis [54] or cell cycle arrest, becoming quiescent [55,56]. The nervous system formed during embryogenesis controls larval functions, while the quiescent NBs are used for its extensive remodeling during metamorphosis [57,58,59,60,61,62]. *Drosophila* glia also play critical roles in developmental axon, dendrite and neurite pruning (reviewed in [63,64]) and in experience-dependent synaptic remodeling (reviewed in [65,66]).

There are multiple types of neuroblasts in larval brains that contribute to adult neuronal and glial populations: Type I and Type II neuroblasts and mushroom body neuroblasts (MBNBs) contribute to the central brain while the optic lobes are derived from other specialized neuroblasts [67,68,69,70]. Type I and Type II neuroblasts, MBNBs, and some optic lobe neuroblasts express the *deadpan* (*dpn*) gene. *dpn* encodes a basic helix–loop–helix (bHLH) transcription factor required for transcriptional repression during neuroblast development. Type I neuroblasts also express *asense* (*ase*), which encodes a bHLH transcription factor that promotes the transition of NBs into GMCs [71]. Hence, *dpn* and *ase* are both involved in nervous system development and cell fate determination. Most neuroblasts exit the cell cycle about 20–30 h post-puparium formation, with final divisions regulated by ecdysone signaling and the Mediator complex [72].

In contrast, the four MBNBs per brain hemisphere continue to produce progeny until 85–90 h post-puparium formation [73] due to extended IGF-II mRNA-binding protein (Imp) expression that inhibits components of the Mediator complex [74]. Under certain conditions, MBNBs can persist into adulthood [75]. MBNBs can be visualized via expression of *tailless* (*tll*) which encodes a nuclear receptor required for proliferation and maintenance of MBNBs and GMCs [76]. The relatively late termination of cell division led to the proposal that the mushroom body exhibits injury-induced activation of adult neurogenesis because it possesses a younger population of quiescent differentiated cells [77]. Intriguingly, the mushroom body functions in olfactory learning and memory, similar to the human hippocampus, where there is also evidence for adult neurogenesis.

### 3.1. Homeostatic Neurogenesis and Gliogenesis in Adult Drosophila Brains

It was widely accepted that the adult *Drosophila* brain was devoid of cell proliferation and that both neurogenesis and gliogenesis are completed during development [73,78]. The observation that glia in the adult *Drosophila* brain proliferate in response to programmed cell death provided early hints that some cells in the adult brain retain proliferative potential [79]. Cell proliferation assays with bromodeoxyuridine (BrdU) labeling and clonal analysis subsequently revealed homeostatic cell proliferation in adult brains [78,80]. The majority the BrdU labeled cells and clones clustered on the ventrolateral side of the antennal lobes. These cells were predominantly glia as evidenced by expression of the glial protein Reversed polarity (Repo) [78]. Using an improved lineage tracing method (perma-twin) to detect adult neurogenesis in the medulla cortex of the adult optic lobes, it then was shown that new neurons also could be generated in the adult brain [81]. These adult-born neurons express the neuronal protein Embryonic lethal, abnormal vision (Elav) and have projections extending into the neuropil [81]. The new neurons were shown to arise from cells expressing the canonical NB protein Dpn. Intriguingly, Dpn was predominantly cytoplasmic in uninjured adult brains, leading the authors to propose that this was indicative of quiescence. Consistent with this, Dpn became nuclear in dividing NBs after injury [81]. Evidence for the existence of proliferative glial cells and putative quiescent neural progenitors has prompted other studies using injury as a tool to induce a regenerative response in the adult brain.

### 3.2. Larval and Adult Drosophila Neural Regeneration Models

In the subsequent sections, we explore the key *Drosophila* models of regeneration in the central and peripheral nervous systems. Most of these models focus on the regrowth of damaged axons and/or dendrites while a few focus on the generation of new neurons and/or glial cells.

#### 3.2.1. *Drosophila* Larval Nerve Crush

**Figure 1:**
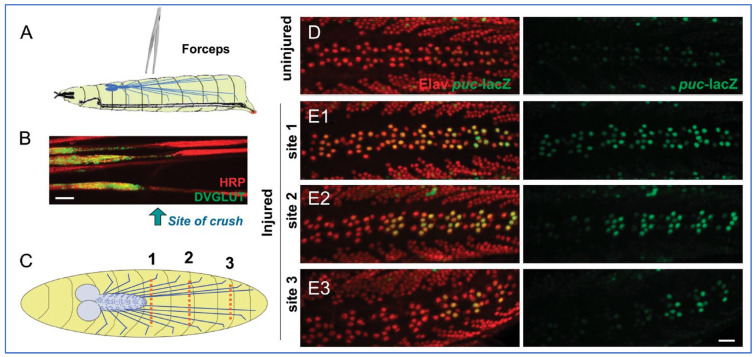
***Drosophila* larval nerve crush.** (**A**) Schematic of the nerve crush assay. The segmental nerves within a third instar larva are crushed by pinching the ventral cuticle with forceps. (**B**) Injured segmental nerves 24 h after nerve crush. Synaptic vesicle precursors detected by staining for DVGLUT staining (green) accumulate at the proximal side of the crush site (arrow). (**C**) Schematic of larva showing brain with neuron cell bodies (blue dots), segmental nerves (blue lines), and different crush sites (numbered 1–3 and indicated with dashed red lines). Site 1 injures more cells than sites 2 and 3 and therefore results in more JNK phosphatase *puckered* expression, reported by a *puc-lacZ* enhancer trap (green) in sample (**E1**) than in control uninjured (**D**), site 2 injured (**E2**) or site 3 injured (**E3**) larvae. *puc-lacZ* is barely detectable in uninjured animals (**D**) and induced in defined subsets of motoneurons around sites of injury (**E1**–**E3**). Neuronal nuclei are stained using anti-Elav in red (**D**–**E3**). Scale bar = 25 μm. (from [82]).

Larval nerve crush models have been helpful for observing axonal responses to injury, including cell signaling mechanisms. This model targets the segmental nerves of *Drosophila*, which contain both sensory and motor axons. Damaging the larval segmental nerves leads to paralysis of the posterior segments; however, the larvae are still able to feed and survive through pupation to eclose into adults with full mobility [82] (Figure 1). Remarkably, 70–80% of injured axons show signs of regenerative growth within 14 h of injury [82]. Soon after injury, the nerves become thin, appear stretched, and are not wide enough for the passage of vesicular cargoes leading to complications from vesicular accumulation [82]. The c-Jun N-terminal kinase (JNK) signaling pathway is implicated in this neural injury response upregulating glial phagocytosis and protecting axons from degeneration. Specifically, axonal injury activates the MAPKKK Wallenda (Wnd), initiating the JNK signaling pathway cascade. The E3 ubiquitin ligase Highwire then feeds back to inhibit Wnd. Not only do increases in Wnd activity protect against axonal degeneration [83], but increased Wnd levels and concomitant decreases in Highwire levels are associated with more rapid axon regeneration [82]. Consistent with this, Wnd has been implicated in neurite sprouting of cultured mushroom body neurons [84] while interactions between JNK signaling and *Drosophila* amyloid precursor protein-like (APPL) promote post-developmental neurite arborization in the adult brain [85].

Injury models in rodents subsequently were found to recapitulate certain responses of injured *Drosophila* axons. Specifically, the mammalian ortholog of *highwire*, Phr1, plays a pivotal role in Wallerian degeneration, a process in which injured axons separate from the soma and undergo fragmentation of cytoskeleton and membrane [86]. In mice where Phr1 was knocked out, Wallerian degeneration was delayed [87]. Loss of Phr1 also increases axonal levels of nicotinamide mononucleotide adenyltransferase 2 (NMNAT2), an axon survival molecule necessary for Phr1-dependent axon stability [87].

#### 3.2.2. *Drosophila* Larval Neurite Ablation

**Figure 2:**
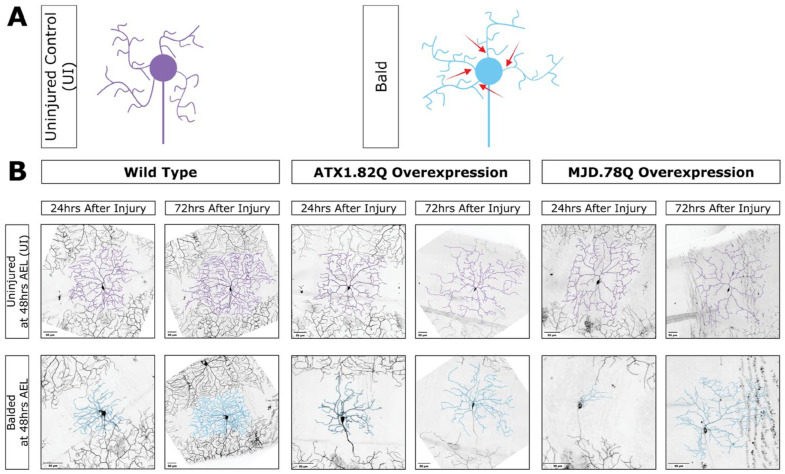
***Drosophila* larval neurite ablation.** (**A**) Schematic of uninjured neurons (purple) and bald neurons (blue). Red arrows represent sites of 2-photon laser injury. (**B**) Uninjured neurons and balded neurons for WT, ATX1.82 overexpression, and MJD.78Q overexpression neurons at 24 and 72 h after injury done at 48 h AEL. Scale bar 50 μm. Neurons overexpressing pathogenic polyQ proteins can regenerate dendrite arbors following complete removal at 48 h AEL (from [89]).

While larval crush assays target the segmental nerves and damage the muscles and cuticle, making them comparable to many human neural injuries, laser ablation is a more precise injury method for dendrites or axons. Dendrites typically receive neural stimuli, while axons signal to other cells. In neurodegenerative diseases like Parkinson’s disease (PD) and Huntington’s disease (HD), dendrites and axons thin and degenerate over time (reviewed in [88]). Remarkably, in a *Drosophila* dendrite ablation model, sensory neurons regenerated the same number of dendrites found in age-matched uninjured neurons at identical developmental time points [89] (Figure 2). Specifically, when all dendrite branches of dendritic arborization (da) neurons were severed (‘balded’) 48 h after egg laying, wild type larvae regenerated their dendritic branches between 24 and 72 h of injury [89]. Dendrite regeneration also gave rise to a neuroprotective response that prevented further neurodegeneration [89]. The response involves early stabilization of the actin cytoskeleton and is specific to dendrites as injury of axons or the cell body does not induce the response [89]. Laser ablations to larval axons and dendrites revealed the mouse protein Wld^s^ (Wallerian degeneration slow) to be neuroprotective when expressed in *Drosophila*, similar to its activity in mammals [90,91]. Wld^s^ is a naturally occurring mutation that gives rise to a fusion protein of Ube4b, a ubiquitination enzyme and Nmnat1, an enzyme involved in making NAD+ (reviewed in [92]).

#### 3.2.3. *Drosophila* Adult Nerve Crush

**Figure 3:**
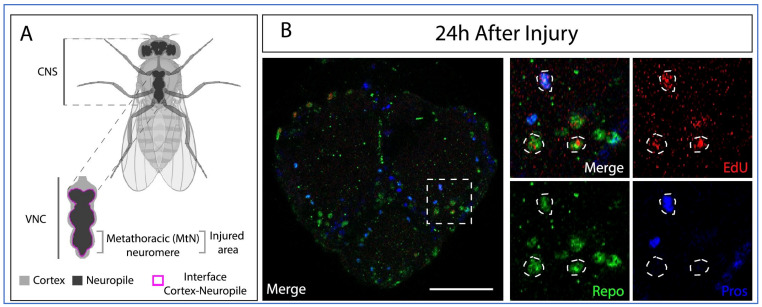
***Drosophila* adult nerve crush.** (**A**) Schematic of the *Drosophila* adult central nervous system (CNS) and ventral nerve cord (VNC), indicating the metathoracic neuromere (MtN), where the injury is performed. (**B**) Representative confocal image with boxed area enlarged in right panels showing dividing cells (dotted circles; EdU^+^) with astrocyte-like glia (ALG) identity (Repo^+^ and Pros^+^) and non-ALG glial identity (Repo^+^, Pros^−^). Scale bar = 50 μm. (from [101]).

The adult nerve crush injury model in *Drosophila* uses forceps to induce nerve damage in the metathoracic segment of adult flies, leading to loss of T3 leg function and impaired locomotion [93]. This injury triggers glial cell proliferation in adult *Drosophila* and results in functional recovery of locomotor activity. One remaining question is what types of glial cells are activated by crush injury. Glial cells in *Drosophila* carry out multiple roles in support of neurons, including metabolic functions [94], forming the blood–brain-barrier (BBB), acting as phagocytes [95,96], and participating in synaptic remodeling [97]. Understanding the molecular and cellular mechanisms used in these disparate activities is crucial for development of therapeutics. There are two subtypes of neuropil-associated glia (NPG), astrocyte-like glia (ALG) and ensheathing glia (EG) [96,98,99,100]. ALG have been shown to contribute to central nervous system (CNS) repair [93], and ALG, but not EG, undergo division following adult nerve crush injury [101] (Figure 3). However, both ALG and EG can generate new neurons through transdifferentiation after damage [101].

#### 3.2.4. *Drosophila* Adult Wing Nerve Ablation

**Figure 4:**
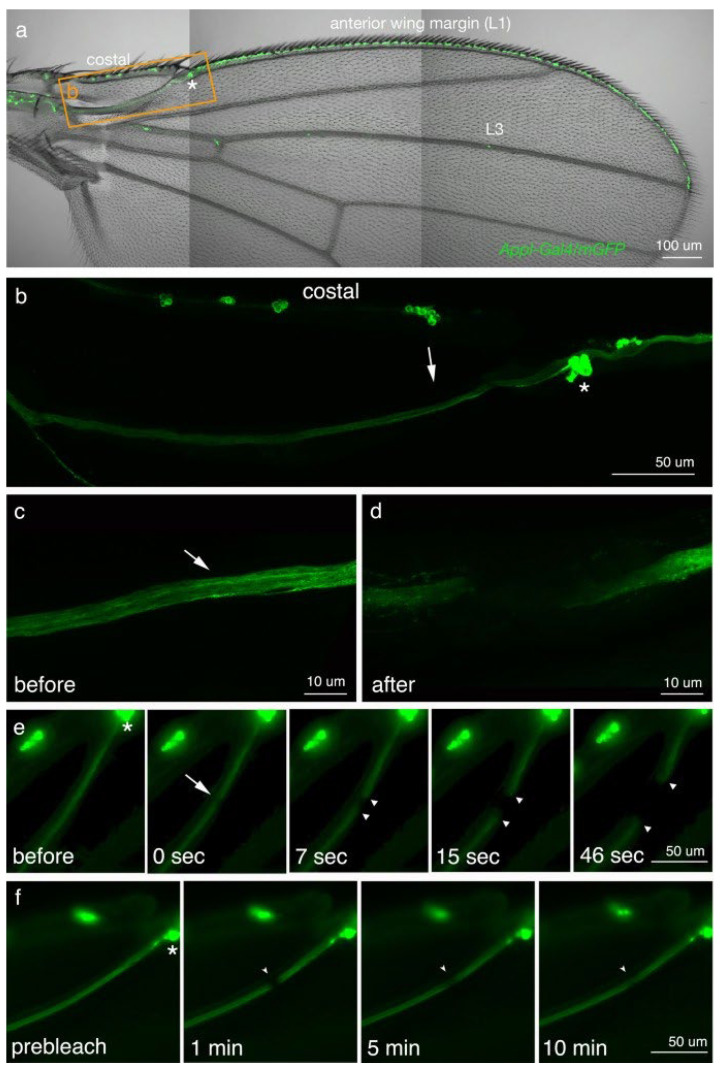
***Drosophila* adult wing nerve ablation.** (**a**) The nerve tract along the wing margin (L1), costal vein, and L3 are highlighted by mCD8-GFP using an appl-GAL4 driver. The image is oriented with the wing tip to the right and the hinge region connected to the body to the left. The wing margin contains mechanosensory and chemosensory neurons from the point of intersection with the costal vein (asterisk) to the intersection with the tip of L3. The neurons send axonal projections in a nerve tract to the thorax. (**b**) Higher magnification of the region in the orange box in panel (**a**) showing the nerve region where axotomy is performed. The last cluster of cells on the costal vein was used as a landmark to position the point of ablation (arrow). The last neural cell bodies along the wing margin are marked (asterisk). (**c**) High magnification of the nerve before laser ablation and (**d**) the same region after ablation. The nerve tract is precisely and completely severed with no visible damage to the surrounding tissue. The region targeted by the laser is indicated with an arrow in (**b**). (**e**) Time-course still images of the ablated nerve. The images are oriented with the first cell bodies of the wing margin on the top right (asterisk) and the nerve tract projecting to the thorax on the bottom left. The laser is centered (0 s, arrow) using the last neural cell bodies on the costal vein (top left) as a landmark to ensure reproducibility. The laser is fired manually until the entire track is cut. Upon complete transection of the wing nerve tract, the two ends separate forming a gap (arrowheads, 7, 15, 46 s). (**f**) The fast recovery of GFP after photobleaching without nerve severing. Asterisk marks the last cell bodies of the wing margin. The region indicated by the arrowhead was photobleached, and subsequent recovery of fluorescence was recorded at 1 min intervals out to 10 min. (from [104]).

The *Drosophila* wing is a powerful model for axon regeneration because its structure permits live visualization of wing vein axon regeneration trajectories using fluorescent protein markers. Wing axon ablation simulates TBI-induced axonal injury while maintaining the cell bodies and proximal axons, thus permitting analysis of axon-specific regeneration [102]. The first longitudinal (L1) wing vein has a nerve with cell bodies spaced along the vein and axons projecting into the CNS. Following ablation and Wallerian-like degeneration of the distal portion of the axon, activated glial cells clear the debris in preparation for regeneration [103]. Furthermore, in young adult (<6 h post-eclosion) *Drosophila* expressing green fluorescent protein (GFP) in their neurons, 50% of the flies with laser-ablated wing axons exhibited axon regeneration by Day 7 [104] (Figure 4). In older flies, injured on Day 3 post-eclosion, axon degeneration was more widespread, but there was still significant regrowth over longer periods of time, suggesting that flies retain some regenerative abilities even as they age [104].

In addition to axon regeneration, scar formation was also observed in adult wings following ablation. Injection of fluorescently labeled dextran (3 kDa) into the wing failed to penetrate the scar, suggesting the scar may act as a physical barrier to axon regrowth [105]. Scar formation in the mammalian CNS limits axon regeneration, possibly due to the scar forming a physical barrier [106,107] and/or due to the production by astrocytes of chondroitin sulfate proteoglycans (CSPGs) that have been shown to inhibit axon growth in vitro (reviewed in [108]). It is possible glial scars function similarly in *Drosophila*. However, more recent work suggests that astrocytes within mammalian scar tissue can also support the regrowth of axons through a lesion [109]. RNA-seq analysis of astrocytes and non-astrocyte cells from spinal cord injury (SCI) lesions show that although they do express many axon inhibitory molecules, they also express axon growth-promoting factors such as laminins, syndecans, and decorin (reviewed in [108]). It may also be the case in *Drosophila*.

The JNK pathway is involved in scar formation and axon degeneration, although its role is complex in mammals, and it has also been implicated in nerve regeneration. In injured mammalian axons, retrograde JNK transport is thought to alter transcription of the injury-response molecule ATF3 and to increase phosphorylation of c-Jun, both of which are important for axonal outgrowth, supporting a pro-regenerative function [110,111,112]. JNK is also activated in damaged mammalian axons near an SCI and its inhibition decreases axonal degeneration [112]. In contrast, studies in *Drosophila* found that expression of a dominant negative form of JNK increased axon regeneration [82,105]. This differs from other regeneration models, such as in the motor neurons of *Drosophila* larvae, and could indicate that JNK has a non-cell autonomous role in regeneration and scar formation, as opposed to an intrinsic role [105].

#### 3.2.5. *Drosophila* Adult Brain Axon Severing

An ex vivo adult *Drosophila* brain model was developed to investigate CNS repair [113]. This model utilizes the precise severing of small lateral neurons ventral (sLNv) axons in adult brain explants, revealing that these neurons possess extremely limited axon regenerative potential. However, the axon regrowth can be enhanced significantly with specific genetic manipulations, including increases in phosphokinase A (PKA) or JNK signaling. In addition, growth of new axons past the lesion site and into the correct target area was also seen with JNK activation [113].

#### 3.2.6. *Drosophila* Adult Penetrating Traumatic Brain Injury

**Figure 5:**
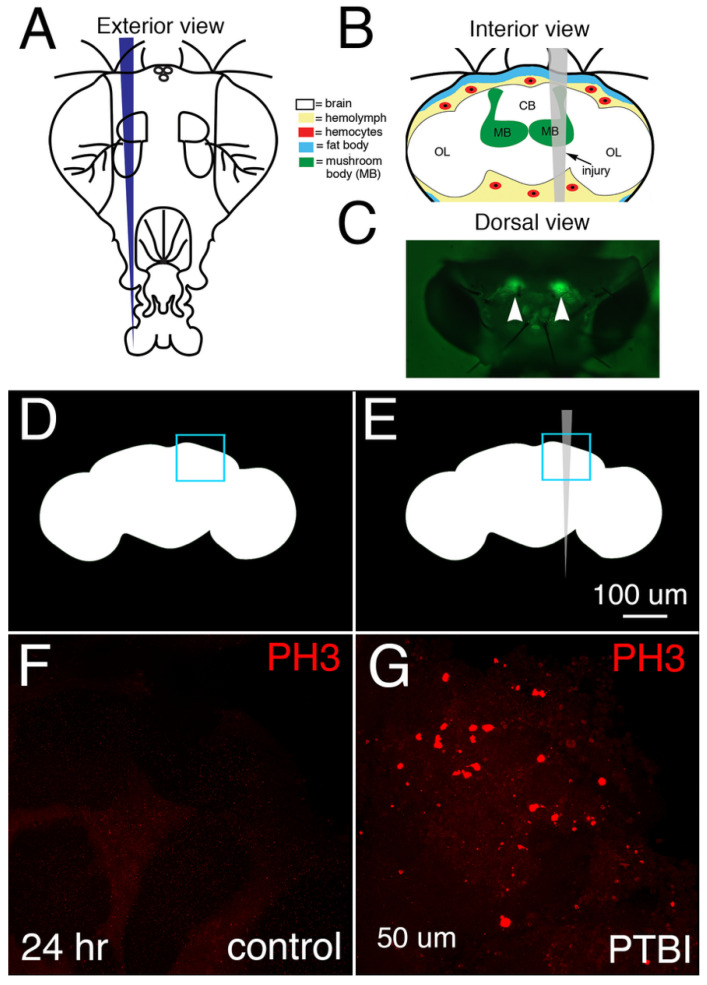
***Drosophila* adult penetrating traumatic brain injury.** (**A**) Schematic of the exterior of an adult fly head. This is a frontal view. Thus, the right side of the animal is to the viewer’s left. (**B**) Schematic of the interior of an adult *Drosophila* head with the injury trajectory indicated in gray. This is a posterior view. Thus, in this image and subsequent figures, the right side of the brain is to the right. Central brain PTBI impacts multiple brain structures including the mushroom body (MB, green), and tissues outside the brain including the fat body (blue) and hemocytes (red). CB = central brain region. OL = optic lobe region. (**C**) Dorsal view of a live adult head in which mushroom bodies (arrowheads) are labeled with green fluorescent protein (GFP). This is our ‘standard genotype’ (see text for details). The PTBI protocol reproducibly results in injury to the mushroom bodies. Uninjured, control (**D**) and PTBI (**E**) schematics. The blue boxes in the upper right corners indicate the brain regions shown at higher magnification in panels (**F**,**G**). (**F**,**G**) PH3 antibody (red) was used to assay for cell proliferation 24 h after injury. In control brains (**F**) there are few PH3^+^ cells, and none near the MB. However, in PTBI brains (**G**), there are PH3^+^ cells near the MB (from [77]).

Penetrating traumatic brain injury (PTBI) to either the optic lobes [81,85,114,115] or the central brain [77,116,117] (Figure 5) has been used to stimulate gliogenesis and neurogenesis. In this model, a thin needle is used to create an injury, and the brains are dissected at various times post-PTBI and imaged with confocal microscopy to visualize proliferating cells. After a PTBI, there is a significant increase in cell proliferation. Dividing cells have been labeled with 5-ethynyl-2′-deoxyuridine (EdU) [77,81,117] which is incorporated into newly synthesized DNA or anti-phosphohistone H3 (anti-pH3) [77,81,114,115,116,117] which transiently marks cells in the G2 and M phases of the cell cycle.

In the optic lobes, it was reported that new neurons were produced following PTBI [81]. It was found later that new glial cells also could be generated in the optic lobes following PTBI [114,115]. Dpn-positive cells were detected in the optic lobes [81] and thought to be quiescent neural progenitors that are activated following injury [115]. The localization of the Dpn protein shifts from predominantly cytoplasmic in undamaged brains to primarily nuclear in injured brains, potentially reflecting a transition from quiescence to a proliferative state [81]. The injury response in the *Drosophila* optic lobes indicated that there is potential for adult neurogenesis and sparked interest in the possibility of similar proliferation in other parts of the adult *Drosophila* brain.

In the central brain, the production of both new neurons and new glia has also been observed in response to injury, with new glia appearing first and arising from preexisting glia [77]. The origins of new neurons in the central brain remain unknown, but they appear to arise from cells that transiently activate *dpn*. Together, these studies make *dpn* a gene of particular interest in *Drosophila* brain regeneration. In the central brain, PTBI-stimulated cell proliferation requires innate immunity pathways [116]. In the optic lobes, reactive oxygen species (ROS) produced by glia following PTBI are thought to stimulate cell proliferation via JNK signaling [114]. The role of ROS following PTBI has not yet been studied in the central brain, nor has the role of innate immune signaling in cell proliferation following PTBI been examined in the optic lobes, although innate immunity genes are upregulated in the optic lobes following PTBI [118].

## 4. Conclusions and Unanswered Questions

In this review, we examine the evidence for regeneration across *Drosophila* nervous system models, specifically crush models, axon and dendrite ablations, and penetrating traumatic brain injuries. Although findings from these models indicate that *Drosophila* can regenerate neurites, glia, and neurons after injury, significant questions remain. Among these are the cellular origins of newborn glia and neurons in the adult brain after injury. While there is strong evidence new glia arise from preexisting glia in both the central brain and the optic lobes, it is unknown whether there is a specific pool of glia capable of cell cycle reentry. Another unresolved question is the origin of adult-born neurons. In the optic lobes, new neurons arise from quiescent *dpn*-expressing NBs. However, in the central brain, this has not been observed. Future studies utilizing live imaging during regeneration could address this question by allowing proliferating cells to be tracked as they repair injuries. Other approaches such as single-cell sequencing coupled with pseudotime and RNA velocity algorithms could help predict the future identity of individual cells and provide clues to the molecular mechanisms underlying neural regeneration. Although the lack of adaptive immunity and a vascular network impose limits on how closely *Drosophila* can model human neurodegenerative disorders, decoding the fundamental molecular mechanics of neural repair in *Drosophila* may allow researchers to identify therapeutic targets and unlock regenerative potential in the human brain.

## Data Availability

No new data were created or analyzed in this study. Data sharing is not applicable to this article.

## References

[1] Ney J.P., Steinmetz J.D., Anderson-Benge E., Gillespie C.W., Becker A., Steele X., Esper G.J. (2026). US Burden of Disorders Affecting the Nervous System: From the Global Burden of Disease 2021 Study. JAMA Neurol..

[2] Gustavsson A., Norton N., Fast T., Frolich L., Georges J., Holzapfel D., Kirabali T., Krolak-Salmon P., Rossini P.M., Ferretti M.T. (2023). Global estimates on the number of persons across the Alzheimer’s disease continuum. Alzheimer’s Dement..

[3] Wang S., Che Y., Lin Y., Zhang Y., He W., Zhang W. (2026). Epidemiology of Parkinson’s disease—Global burden of disease research from 1990 to 2021 and future trend predictions. Clin. Park Relat. Disord..

[4] Yang R., Sun M., Chen W., Feng H., Chen B., Liu Y., He Q., Wang L., Zou C., Luo X. (2026). Global, regional and national burden of Parkinson’s disease, 1990–2021: Update from the GBD 2021 study. J. Neurol. Sci..

[5] Zhong H., Feng Y., Shen J., Rao T., Dai H., Zhong W., Zhao G. (2025). Global Burden of Traumatic Brain Injury in 204 Countries and Territories From 1990 to 2021. Am. J. Prev. Med..

[6] Long K., Huang E., Yuan Y., Wang S., Rui M. (2025). Steering Axon Development: Glial Cell Mechanisms in *Drosophila*. ACS Chem. Neurosci..

[7] Parvez F., Rahul (2026). Immune crosstalk in Alzheimer’s and Parkinson’s disease: Insights from *Drosophila* models into the brain-peripheral immune axis. Front. Immunol..

[8] Lucini C., Gatta C. (2025). Glial Diversity and Evolution: Insights from Teleost Fish. Brain Sci..

[9] Rusznak Z., Henskens W., Schofield E., Kim W.S., Fu Y. (2016). Adult Neurogenesis and Gliogenesis: Possible Mechanisms for Neurorestoration. Exp. Neurobiol..

[10] Bolus H., Crocker K., Boekhoff-Falk G., Chtarbanova S. (2020). Modeling Neurodegenerative Disorders in *Drosophila melanogaster*. Int. J. Mol. Sci..

[11] Gujar M.R., Wang H. (2025). Signaling mechanisms in the reactivation of quiescent neural stem cells in *Drosophila*. Curr. Opin. Cell Biol..

[12] Li G., Hidalgo A. (2020). Adult Neurogenesis in the *Drosophila* Brain: The Evidence and the Void. Int. J. Mol. Sci..

[13] Liu X., Zhao Y., Zou W. (2023). Molecular mechanisms of neurite regeneration and repair: Insights from *C. elegans* and *Drosophila*. Cell Regen..

[14] Otsuki L., Brand A.H. (2020). Quiescent Neural Stem Cells for Brain Repair and Regeneration: Lessons from Model Systems. Trends Neurosci..

[15] Richardson C.E., Shen K. (2019). Neurite Development and Repair in Worms and Flies. Annu. Rev. Neurosci..

[16] Seifert A.W., Duncan E.M., Zayas R.M. (2023). Enduring questions in regenerative biology and the search for answers. Commun. Biol..

[17] Bier E. (2005). *Drosophila*, the golden bug, emerges as a tool for human genetics. Nat. Rev. Genet..

[18] Shin M., Copeland J.M., Venton B.J. (2018). *Drosophila* as a Model System for Neurotransmitter Measurements. ACS Chem. Neurosci..

[19] Davis R.L., Han K.A. (1996). Neuroanatomy: Mushrooming mushroom bodies. Curr. Biol..

[20] Ou J., Gao Z., Song L., Ho M.S. (2016). Analysis of Glial Distribution in *Drosophila* Adult Brains. Neurosci. Bull..

[21] von Bartheld C.S., Bahney J., Herculano-Houzel S. (2016). The search for true numbers of neurons and glial cells in the human brain: A review of 150 years of cell counting. J. Comp. Neurol..

[22] Dorkenwald S., Matsliah A., Sterling A.R., Schlegel P., Yu S.C., McKellar C.E., Lin A., Costa M., Eichler K., Yin Y. (2024). Neuronal wiring diagram of an adult brain. Nature.

[23] Schlegel P., Yin Y., Bates A.S., Dorkenwald S., Eichler K., Brooks P., Han D.S., Gkantia M., Dos Santos M., Munnelly E.J. (2024). Whole-brain annotation and multi-connectome cell typing of *Drosophila*. Nature.

[24] Herculano-Houzel S. (2009). The human brain in numbers: A linearly scaled-up primate brain. Front. Hum. Neurosci..

[25] Hirth F., Reichert H. (1999). Conserved genetic programs in insect and mammalian brain development. Bioessays.

[26] Bellen H.J., Tong C., Tsuda H. (2010). 100 years of *Drosophila* research and its impact on vertebrate neuroscience: A history lesson for the future. Nat. Rev. Neurosci..

[27] Weng M., Lee C.Y. (2011). Keeping neural progenitor cells on a short leash during *Drosophila* neurogenesis. Curr. Opin. Neurobiol..

[28] Herrera S.C., Bach E.A. (2019). JAK/STAT signaling in stem cells and regeneration: From *Drosophila* to vertebrates. Development.

[29] Staley B.K., Irvine K.D. (2012). Hippo signaling in *Drosophila*: Recent advances and insights. Dev. Dyn..

[30] Ramon y Cajal S. (1913). Estudios sobre la degeneracion y regeneracion del sistema nervioso. Tomo I, Degeneracio´n y regeneracio´n de los nervios.

[31] Ramon y Cajal S. (1914). Estudios sobre la degeneracion y regeneracion del sistema nervioso. Tomo II, Degeneracion y regeneracion de los centros nerviosos.

[32] Altman J. (1969). Autoradiographic and histological studies of postnatal neurogenesis. 3. Dating the time of production and onset of differentiation of cerebellar microneurons in rats. J. Comp. Neurol..

[33] Altman J. (1969). Autoradiographic and histological studies of postnatal neurogenesis. IV. Cell proliferation and migration in the anterior forebrain, with special reference to persisting neurogenesis in the olfactory bulb. J. Comp. Neurol..

[34] Altman J., Das G.D. (1965). Autoradiographic and histological evidence of postnatal hippocampal neurogenesis in rats. J. Comp. Neurol..

[35] Altman J., Das G.D. (1966). Autoradiographic and histological studies of postnatal neurogenesis. I. A longitudinal investigation of the kinetics, migration and transformation of cells incorporating tritiated thymidine in neonate rats, with special reference to postnatal neurogenesis in some brain regions. J. Comp. Neurol..

[36] Kaplan M.S., Bell D.H. (1984). Mitotic neuroblasts in the 9-day-old and 11-month-old rodent hippocampus. J. Neurosci..

[37] Kriegstein A., Alvarez-Buylla A. (2009). The glial nature of embryonic and adult neural stem cells. Annu Rev. Neurosci..

[38] Kuhn H.G., Dickinson-Anson H., Gage F.H. (1996). Neurogenesis in the dentate gyrus of the adult rat: Age-related decrease of neuronal progenitor proliferation. J. Neurosci..

[39] Eriksson P.S., Perfilieva E., Bjork-Eriksson T., Alborn A.M., Nordborg C., Peterson D.A., Gage F.H. (1998). Neurogenesis in the adult human hippocampus. Nat. Med..

[40] Sharma K., Agrawal D., Gabrani R. (2026). A patent review on traumatic brain injuries (2020–2025). Int. J. Neurosci..

[41] Yan X., Shen H., Zhao M., Nie S., Huang Y., Sun J. (2026). Time Is of the Essence: Temporal Dynamics of Epigenetic Landscapes as Therapeutic Targets in Traumatic Brain Injury. ACS Chem. Neurosci..

[42] Fesharaki-Zadeh A. (2022). Oxidative Stress in Traumatic Brain Injury. Int. J. Mol. Sci..

[43] Ardic N., Dinc R. (2026). Neutrophil extracellular traps and microglia/macrophages interactions in stroke: From thromboinflammation to immunotherapy. Front. Immunol..

[44] Ferrazzoli D., Ortelli P., Versace V., Stolz J., Dezi S., Vos P., Giladi N., Saltuari L., Sebastianelli L. (2024). Post-traumatic parkinsonism: The intricate twist between trauma, inflammation and neurodegeneration. A narrative review. J. Neurol. Sci..

[45] Yadav A.K., Verma P., Srivastava A., Srivastava P., Rai R., Rathour S. (2026). Molecular insights into glial neuroimmune cross reactivity with CNS antigens and its role in neuroinflammation. Inflammopharmacology.

[46] Sofroniew M.V. (2009). Molecular dissection of reactive astrogliosis and glial scar formation. Trends Neurosci..

[47] Alhadidi Q.M., Bahader G.A., Arvola O., Kitchen P., Shah Z.A., Salman M.M. (2024). Astrocytes in functional recovery following central nervous system injuries. J. Physiol..

[48] Duan C.L., Liu C.W., Shen S.W., Yu Z., Mo J.L., Chen X.H., Sun F.Y. (2015). Striatal astrocytes transdifferentiate into functional mature neurons following ischemic brain injury. Glia.

[49] Tasdemir-Yilmaz O.E., Freeman M.R. (2014). Astrocytes engage unique molecular programs to engulf pruned neuronal debris from distinct subsets of neurons. Genes Dev..

[50] Ziebell J.M., Morganti-Kossmann M.C. (2010). Involvement of pro- and anti-inflammatory cytokines and chemokines in the pathophysiology of traumatic brain injury. Neurotherapeutics.

[51] Carmena A. (2020). The Case of the Scribble Polarity Module in Asymmetric Neuroblast Division in Development and Tumorigenesis. Int. J. Mol. Sci..

[52] Maurange C. (2020). Temporal patterning in neural progenitors: From *Drosophila* development to childhood cancers. Dis. Model Mech..

[53] Mira H., Morante J. (2020). Neurogenesis From Embryo to Adult—Lessons From Flies and Mice. Front. Cell Dev. Biol..

[54] Abrams J.M., White K., Fessler L.I., Steller H. (1993). Programmed cell death during *Drosophila* embryogenesis. Development.

[55] Ebens A.J., Garren H., Cheyette B.N., Zipursky S.L. (1993). The *Drosophila* anachronism locus: A glycoprotein secreted by glia inhibits neuroblast proliferation. Cell.

[56] Edgar B.A., O’Farrell P.H. (1990). The three postblastoderm cell cycles of *Drosophila* embryogenesis are regulated in G2 by string. Cell.

[57] Lee T., Lee A., Luo L. (1999). Development of the *Drosophila* mushroom bodies: Sequential generation of three distinct types of neurons from a neuroblast. Development.

[58] Marin E.C., Watts R.J., Tanaka N.K., Ito K., Luo L. (2005). Developmentally programmed remodeling of the *Drosophila* olfactory circuit. Development.

[59] Sood C., Doyle S.E., Siegrist S.E. (2021). Steroid hormones, dietary nutrients, and temporal progression of neurogenesis. Curr. Opin. Insect Sci..

[60] Technau G., Heisenberg M. (1982). Neural reorganization during metamorphosis of the corpora pedunculata in *Drosophila melanogaster*. Nature.

[61] Truman J.W., Riddiford L.M. (2023). *Drosophila* postembryonic nervous system development: A model for the endocrine control of development. Genetics.

[62] Truman J.W., Schuppe H., Shepherd D., Williams D.W. (2004). Developmental architecture of adult-specific lineages in the ventral CNS of *Drosophila*. Development.

[63] Furusawa K., Emoto K. (2021). Spatiotemporal regulation of developmental neurite pruning: Molecular and cellular insights from *Drosophila* models. Neurosci. Res..

[64] Yu F., Schuldiner O. (2014). Axon and dendrite pruning in *Drosophila*. Curr. Opin. Neurobiol..

[65] Miller V.K., Broadie K. (2026). Partners in plasticity: Serotonergic glial interactions in brain circuit remodeling. Front. Neurosci..

[66] Nelson N., Miller V., Broadie K. (2025). Neuron-to-glia and glia-to-glia signaling directs critical period experience-dependent synapse pruning. Front. Cell Dev. Biol..

[67] Cabernard C. (2026). Asymmetry and the cytoskeleton: Mechanisms of asymmetric neural stem cell division in *Drosophila melanogaster*. Curr. Top. Dev. Biol..

[68] Homem C.C., Knoblich J.A. (2012). *Drosophila* neuroblasts: A model for stem cell biology. Development.

[69] Homem C.C., Repic M., Knoblich J.A. (2015). Proliferation control in neural stem and progenitor cells. Nat. Rev. Neurosci..

[70] Leclercq J., Maurange C. (2025). From the Making of a Neural Lineage to the Making of a Tumor: Lessons from the “Simple” *Drosophila* Brain. Adv. Exp. Med. Biol..

[71] Soares D.S., Homem C.C.F., Castro D.S. (2022). Function of Proneural Genes Ascl1 and Asense in Neurogenesis: How Similar Are They?. Front. Cell Dev. Biol..

[72] Homem C.C.F., Steinmann V., Burkard T.R., Jais A., Esterbauer H., Knoblich J.A. (2014). Ecdysone and mediator change energy metabolism to terminate proliferation in *Drosophila* neural stem cells. Cell.

[73] Ito K., Hotta Y. (1992). Proliferation pattern of postembryonic neuroblasts in the brain of *Drosophila melanogaster*. Dev. Biol..

[74] Yang C.P., Samuels T.J., Huang Y., Yang L., Ish-Horowicz D., Davis I., Lee T. (2017). Imp and Syp RNA-binding proteins govern decommissioning of *Drosophila* neural stem cells. Development.

[75] Siegrist S.E., Haque N.S., Chen C.H., Hay B.A., Hariharan I.K. (2010). Inactivation of both Foxo and reaper promotes long-term adult neurogenesis in *Drosophila*. Curr. Biol..

[76] Kurusu M., Maruyama Y., Adachi Y., Okabe M., Suzuki E., Furukubo-Tokunaga K. (2009). A conserved nuclear receptor, Tailless, is required for efficient proliferation and prolonged maintenance of mushroom body progenitors in the *Drosophila* brain. Dev. Biol..

[77] Crocker K.L., Marischuk K., Rimkus S.A., Zhou H., Yin J.C.P., Boekhoff-Falk G. (2021). Neurogenesis in the adult *Drosophila* brain. Genetics.

[78] von Trotha J.W., Egger B., Brand A.H. (2009). Cell proliferation in the *Drosophila* adult brain revealed by clonal analysis and bromodeoxyuridine labelling. Neural Dev..

[79] Kato K., Awasaki T., Ito K. (2009). Neuronal programmed cell death induces glial cell division in the adult *Drosophila* brain. Development.

[80] Foo L.C., Song S., Cohen S.M. (2017). miR-31 mutants reveal continuous glial homeostasis in the adult *Drosophila* brain. EMBO J..

[81] Fernandez-Hernandez I., Rhiner C., Moreno E. (2013). Adult neurogenesis in *Drosophila*. Cell Rep..

[82] Xiong X., Wang X., Ewanek R., Bhat P., Diantonio A., Collins C.A. (2010). Protein turnover of the Wallenda/DLK kinase regulates a retrograde response to axonal injury. J. Cell Biol..

[83] Xiong X., Collins C.A. (2012). A conditioning lesion protects axons from degeneration via the Wallenda/DLK MAP kinase signaling cascade. J. Neurosci..

[84] Marmor-Kollet N., Schuldiner O. (2016). Contrasting developmental axon regrowth and neurite sprouting of *Drosophila* mushroom body neurons reveals shared and unique molecular mechanisms. Dev. Neurobiol..

[85] Leyssen M., Ayaz D., Hebert S.S., Reeve S., De Strooper B., Hassan B.A. (2005). Amyloid precursor protein promotes post-developmental neurite arborization in the *Drosophila* brain. EMBO J..

[86] Conforti L., Gilley J., Coleman M.P. (2014). Wallerian degeneration: An emerging axon death pathway linking injury and disease. Nat. Rev. Neurosci..

[87] Babetto E., Beirowski B., Russler E.V., Milbrandt J., DiAntonio A. (2013). The Phr1 ubiquitin ligase promotes injury-induced axon self-destruction. Cell Rep..

[88] Kweon J.H., Kim S., Lee S.B. (2017). The cellular basis of dendrite pathology in neurodegenerative diseases. BMB Rep..

[89] Prange S.E., Bhakta I.N., Sysoeva D., Jean G.E., Madisetti A., Le H.H.N., Duong L.U., Hwu P.T., Melton J.G., Thompson-Peer K.L. (2024). Dendrite injury triggers neuroprotection in *Drosophila* models of neurodegenerative disease. Sci. Rep..

[90] Avery M.A., Rooney T.M., Pandya J.D., Wishart T.M., Gillingwater T.H., Geddes J.W., Sullivan P.G., Freeman M.R. (2012). WldS prevents axon degeneration through increased mitochondrial flux and enhanced mitochondrial Ca2+ buffering. Curr. Biol..

[91] Tao J., Rolls M.M. (2011). Dendrites have a rapid program of injury-induced degeneration that is molecularly distinct from developmental pruning. J. Neurosci..

[92] Llobet Rosell A., Neukomm L.J. (2019). Axon death signalling in Wallerian degeneration among species and in disease. Open Biol..

[93] Losada-Perez M., Garcia-Guillen N., Casas-Tinto S. (2021). A novel injury paradigm in the central nervous system of adult *Drosophila*: Molecular, cellular and functional aspects. Dis. Model Mech..

[94] Volkenhoff A., Weiler A., Letzel M., Stehling M., Klambt C., Schirmeier S. (2015). Glial Glycolysis Is Essential for Neuronal Survival in *Drosophila*. Cell Metab..

[95] Cantera R., Technau G.M. (1996). Glial cells phagocytose neuronal debris during the metamorphosis of the central nervous system in *Drosophila melanogaster*. Dev. Genes Evol..

[96] Hakim-Mishnaevski K., Flint-Brodsly N., Shklyar B., Levy-Adam F., Kurant E. (2019). Glial Phagocytic Receptors Promote Neuronal Loss in Adult *Drosophila* Brain. Cell Rep..

[97] Chang Y.C., Peng Y.J., Lee J.Y., Wen A., Chang K.T. (2025). Peripheral glia and neurons jointly regulate activity-induced synaptic remodeling at the *Drosophila* neuromuscular junction. eLife.

[98] Kremer M.C., Jung C., Batelli S., Rubin G.M., Gaul U. (2017). The glia of the adult *Drosophila* nervous system. Glia.

[99] Losada-Perez M. (2018). Glia: From ’just glue’ to essential players in complex nervous systems: A comparative view from flies to mammals. J. Neurogenet..

[100] Shweta, Sharma K., Shakarad M., Agrawal N., Maurya S.K. (2024). *Drosophila* glial system: An approach towards understanding molecular complexity of neurodegenerative diseases. Mol. Biol. Rep..

[101] Casas-Tinto S., Garcia-Guillen N., Losada-Perez M. (2025). Adult neurogenesis through glial transdifferentiation in a CNS injury paradigm. eLife.

[102] Fang Y., Bonini N.M. (2015). Hope on the (fruit) fly: The *Drosophila* wing paradigm of axon injury. Neural Regen. Res..

[103] Fang Y., Bonini N.M. (2012). Axon degeneration and regeneration: Insights from *Drosophila* models of nerve injury. Annu Rev. Cell Dev. Biol..

[104] Soares L., Parisi M., Bonini N.M. (2014). Axon injury and regeneration in the adult *Drosophila*. Sci. Rep..

[105] Brace E.J., Wu C., Valakh V., DiAntonio A. (2014). SkpA restrains synaptic terminal growth during development and promotes axonal degeneration following injury. J. Neurosci..

[106] Fawcett J.W., Asher R.A. (1999). The glial scar and central nervous system repair. Brain Res. Bull..

[107] Ohtake Y., Li S. (2015). Molecular mechanisms of scar-sourced axon growth inhibitors. Brain Res..

[108] Silver J., Miller J.H. (2004). Regeneration beyond the glial scar. Nat. Rev. Neurosci..

[109] Anderson M.A., Burda J.E., Ren Y., Ao Y., O’Shea T.M., Kawaguchi R., Coppola G., Khakh B.S., Deming T.J., Sofroniew M.V. (2016). Astrocyte scar formation aids central nervous system axon regeneration. Nature.

[110] Lindwall C., Kanje M. (2005). The Janus role of c-Jun: Cell death versus survival and regeneration of neonatal sympathetic and sensory neurons. Exp. Neurol..

[111] Lindwall C., Kanje M. (2005). Retrograde axonal transport of JNK signaling molecules influence injury induced nuclear changes in p-c-Jun and ATF3 in adult rat sensory neurons. Mol. Cell Neurosci..

[112] Yoshimura K., Ueno M., Lee S., Nakamura Y., Sato A., Yoshimura K., Kishima H., Yoshimine T., Yamashita T. (2011). c-Jun N-terminal kinase induces axonal degeneration and limits motor recovery after spinal cord injury in mice. Neurosci. Res..

[113] Ayaz D., Leyssen M., Koch M., Yan J., Srahna M., Sheeba V., Fogle K.J., Holmes T.C., Hassan B.A. (2008). Axonal injury and regeneration in the adult brain of *Drosophila*. J. Neurosci..

[114] Alves C.S., Simoes A.R., Gil Ferreira B., Neto M., Soares C.C., Augusto A., Rhiner C. (2026). Duox-driven ROS release by glia promotes regeneration in the adult *Drosophila* brain. EMBO Rep..

[115] Simoes A.R., Neto M., Alves C.S., Santos M.B., Fernandez-Hernandez I., Veiga-Fernandes H., Brea D., Dura I., Encinas J.M., Rhiner C. (2022). Damage-responsive neuro-glial clusters coordinate the recruitment of dormant neural stem cells in *Drosophila*. Dev. Cell.

[116] Ahern-Djamali S., Marischuk K., Crocker K.L., Peetz I., Scott E., Boekhoff-Falk G. (2025). Innate immunity pathways activate cell proliferation after penetrating traumatic brain injury in adult *Drosophila*. Fly.

[117] Crocker K.L., Ahern-Djamali S., Boekhoff-Falk G. (2021). Stimulating and Analyzing Adult Neurogenesis in the *Drosophila* Central Brain. J. Vis. Exp..

[118] Sanuki R., Tanaka T., Suzuki F., Ibaraki K., Takano T. (2019). Normal aging hyperactivates innate immunity and reduces the medical efficacy of minocycline in brain injury. Brain Behav. Immun..

